# Domain Adaptation for Imitation Learning Using Generative Adversarial Network

**DOI:** 10.3390/s21144718

**Published:** 2021-07-09

**Authors:** Tho Nguyen Duc, Chanh Minh Tran, Phan Xuan Tan, Eiji Kamioka

**Affiliations:** 1 Graduate School of Engineering and Science, Shibaura Institute of Technology, Tokyo 135-8548, Japan; nb20501@shibaura-it.ac.jp (T.N.D.); nb20502@shibaura-it.ac.jp (C.M.T.); kamioka@shibaura-it.ac.jp (E.K.); 2 Department of Information and Communications Engineering, Shibaura Institute of Technology, Tokyo 135-8548, Japan

**Keywords:** imitation learning, domain adaptive imitation learning, generative adversarial network

## Abstract

Imitation learning is an effective approach for an autonomous agent to learn control policies when an explicit reward function is unavailable, using demonstrations provided from an expert. However, standard imitation learning methods assume that the agents and the demonstrations provided by the expert are in the same domain configuration. Such an assumption has made the learned policies difficult to apply in another distinct domain. The problem is formalized as domain adaptive imitation learning, which is the process of learning how to perform a task optimally in a learner domain, given demonstrations of the task in a distinct expert domain. We address the problem by proposing a model based on Generative Adversarial Network. The model aims to learn both domain-shared and domain-specific features and utilizes it to find an optimal policy across domains. The experimental results show the effectiveness of our model in a number of tasks ranging from low to complex high-dimensional.

## 1. Introduction

The demand for autonomous agents capable of mimicking human behaviors has grown significantly in recent years. For example, self-driving vehicles, assistive robots, and human–computer interaction fields rely on the ability of agents that can not only make optimal decisions but also behave like humans [1], which can enable the agents’ actions to be believable and appear natural. In order for autonomous agents to acquire such human complex behaviors, they are supplied with reward functions indicating the goals of the desired behaviors. However, reward functions can be difficult to be defined manually. In fact, humans can learn complex behaviors from imitation: we observe other experts performing the tasks, infer the tasks, then attempt to accomplish the same tasks ourselves. Inspired by this learning procedure, imitation learning has been widely used for training autonomous agents using expert-provided demonstrations [1,2,3,4].

Imitation learning works by extracting information about the behavior of the expert and learning a mapping between the observation state and demonstrated behavior [1,5]. Unfortunately, the traditional imitation learning algorithms are still far from being comparable with the human imitation due to the lack of the following abilities:

1. Humans tend to imitate the goal of a task rather than a particular behavior of the expert [6,7].
2. Humans can recognize structural differences (i.e., domain shift) and similarities between the expert and themselves in order to adapt their behaviors accordingly [8].

The first aspect of human imitation can be modeled using Inverse Reinforcement Learning (IRL) [9,10]. IRL seeks to estimate a reward function to explain an expert behavior from demonstrations and subsequently train an agent on it [9,10,11,12]. Recent studies [13,14,15,16,17,18,19,20] utilize Generative Adversarial Network (GAN) [21], which has a discriminator to judge whether a given behavior is from an expert or agent, and then a policy is trained using the discriminator as a reward. However, these approaches do not take into account the second aspect of human learning: imitation with the presence of domain shift between the expert and the agent. Such domain shift can mislead the feature learning, resulting in poor task performance.

The problem is formalized as domain adaptive imitation learning, which is a process of learning how to perform a task optimally in a learner domain, given demonstrations of the task in a distinct expert domain [14]. In order to solve this problem, the authors in [14] proposed a two-step approach: alignment followed by adaptation. Firstly, the Generative Adversarial MDP Alignment (GAMA) was introduced to learn the state—action maps from demonstrations. Then, in the adaptation step, an optimal policy for the learner domain was obtained using the learned alignment from the first step. Despite showing a promising result, their model was evaluated only in low-dimensional tasks. In addition, they updated the learned policy by using behavioral cloning, which was vulnerable to cascading errors. This could lead to poor adaptation performance in more complex high-dimensional tasks.

Unlike most previous studies in domain adaptive imitation learning, this work proposes a model that aims to learn both domain-shared and domain-specific features. Such features enable the agents to learn optimal policies without being affected by the shift between two domains. The learning procedure can be achieved within one training process by utilizing adversarial training [21]. In summary, the main contributions of this paper are as follows:

- A features extractor, which is capable of deriving domain-shared and domain-specific features, is proposed.
- The DAIL-GAN model is proposed. The model leverages adversarial training [21] to learn the extracted features, while at the same time, seeking for an optimal learner domain policy.
- A comprehensive experiment on both low and high-dimensional tasks is conducted to evaluate the performance of the proposed model.

The rest of this paper is organized as follows. In Section 2, the related works of the proposed model is introduced. Section 3 formulates the domain adaptive imitation learning problem. The details of the proposed DAIL-GAN model is presented in Section 4 and evaluated in Section 5. Section 6 discusses and analyzes the evaluation results. Finally, Section 7 concludes this paper.

## 2. Related Work

Imitation learning has been a popular method for training autonomous agents from expert demonstrations [1]. A simple approach to imitation learning is Behavioral Cloning (BC) [22], which mimics such demonstrations by learning the policy through supervised learning. Despite being successfully applied in many control problems [2,22,23], BC was found to be vulnerable to cascading errors [24]. On the other hand, Inverse Reinforcement Learning (IRL) [9] methods try to recover a reward function from the expert demonstrations [9,10,11,12]. This reward function is then used to optimize an imitation policy by running a standard reinforcement learning [25,26]. Accordingly, IRL has succeeded in a wide range of tasks [27,28,29,30]. However, in order to train an IRL model, it requires iterations of reinforcement learning, which can be extremely computationally expensive for high-dimensional tasks. Recently, Generative Adversarial Network [21] has been introduced and successfully employed to tackle complex challenges in image generation, translation, and enhancement [31,32,33,34]. Inspired by the great ability of GAN, recent studies [13,15,16,17,18,19,20] have applied it in imitation learning to define expert behaviors by fitting the distributions of states and actions. These models outperform competing methods when applying to complex high-dimensional tasks over various amounts of expert data.

Unfortunately, the common major weakness of the above-mentioned models is that they require the experts to provide demonstrations in the same configuration and domain as the learners. Thus, the presence of a shift between the expert and learner domains may lead to a significant performance deterioration of those models. A popular approach is to employ a domain adaptation, which attempts to recover the learned policy from one domain and to adapt it to a different domain. The work in [35] proposed a model to recover domain-agnostic features and utilized it to find optimal policies in the setting of third person imitation, in which the expert and learner observations come from different views. Furthermore, the authors in [14] introduced a two-step approach that could be applied to imitate demonstrations observed from a distinct domain. They proposed to find a state–action mapping between the expert and learner domains. After that, the learned mapping was utilized to adapt the learned policy to the learner domain. Although achieving high performance on low-dimensional tasks, the effectiveness of their methods on more complex high-dimensional tasks was not fully inspected yet.

Our method is different from previous methods [14,35], which aims to learn both domain-shared and domain-specific features in expert and learner domains. These features enable our proposed model to find an optimal learner domain policy that can achieve high performance without being affected by the shift between two domains.

## 3. Problem Formulation

In this section, we formalize the domain adaptive imitation learning as a Markov decision problem. A Markov Decision Process (MDP) M with finite time horizon [36] is represented as the following equation:

(1) M=(S,A,P,R)

where S and A represent the state and action space, respectively; P:S×A→S denotes the transition function, and R:S×A→R is the reward function—whereas, a policy π:S→A for M describes a mapping from states S to actions A. In general reinforcement learning setting, the goal is to find an optimal policy $\pi^{*}$ that achieves the highest expected discounted sum of rewards *J*:

(2) $$\begin{array}{cc} \; & \pi^{*}=\underset{\pi }{argmax}J(\pi ), \end{array}$$

(3) $$\begin{array}{cc} \; & \;\mathrm{subject}\;\mathrm{to}\;\;J\left(\pi \right)=E_{\pi }\left[\sum_{t=0}^{T}\gamma^{t}r^{t}\right] \end{array}$$

where γ∈(0,1] is the discount factor and $r^{t}=R\left(s^{t},a^{t}\right)$ is the reward at timestep *t*.

**Figure 1:**
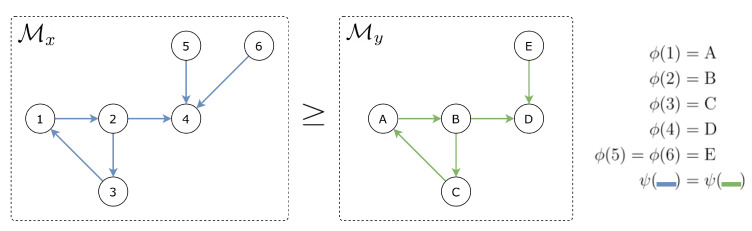
An example of two MDPs for *x* and *y* domains where $M_{x}\geq M_{y}$. $\phi :S_{x}\to S_{y}$ and $\psi :A_{x}\to A_{y}$ are state and actions maps, respectively. States correspond to nodes and actions to colors. States 5, 6 in $S_{x}$ are merged to state *e* in $S_{y}$ and blue actions in $A_{x}$ are mapped to green actions in $A_{y}$.

However, in the domain adaptive imitation learning setting, the reward function is not given beforehand. Therefore, the MDP for a domain *x* without reward is defined as $M_{x}=\left(S_{x},A_{x},P_{x}\right)$. In this paper, all examined domains are assumed to be alignable. That is, if considering two domains *x* and *y*, $M_{x}$ can be reduced to $M_{y}$, denoted as $M_{x}\geq M_{y}$, or vice versa [14]. An example is illustrated in Figure 1. Based on this expression, let E and L be the expert and the learner domain, respectively, $M_{E}$ and $M_{L}$ are said to be alignable if and only if $M_{E}\geq M_{L}$ or $M_{L}\geq M_{E}$ [14].

Furthermore, $\tau_{x}=\left\{\left(s_{x}^{t},a_{x}^{t}\right):t\in \left[0,T\right]\right\}$ denotes a demonstration in the domain *x*, which is a sequence of state–action pairs. Then, a set of demonstrations $D_{E}=\left\{\tau_{E}^{i}:i\in \left[1,N\right]\right\}$ from E is assumed to be available at the training time. With those assumptions, our main objective is being able to learn an optimal learner domain policy $\pi_{L}^{*}$ against unknown reward function $R_{L}$, given the expert demonstrations $D_{E}$.

## 4. The Proposed DAIL-GAN Model

**Figure 2:**
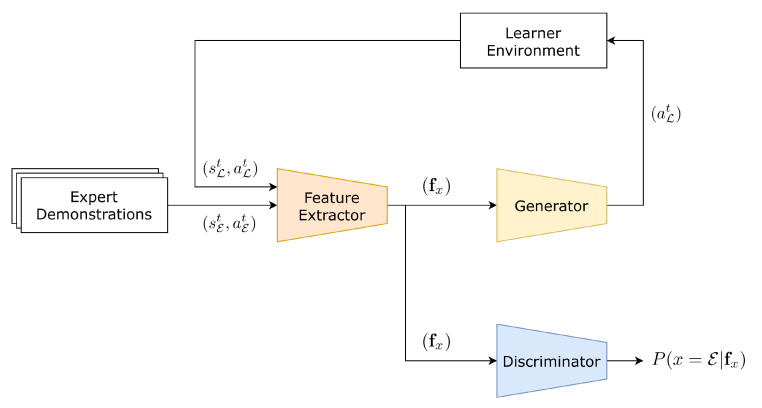
The architecture of the proposed DAIL-GAN model.

In this section, we introduce our proposed DAIL-GAN model. The model relies on learning the domain-shared and domain-specific features in order to recover expert behaviors and adapt them to the learner domain. The architecture of our proposed model is illustrated in Figure 2. The model includes three deep feed-forward networks *F*, *G*, and *D* that holds different responsibilities.

### 4.1. Feature Extractor Network F

A state–action pair $\left(s_{x}^{t},a_{x}^{t}\right)$ in domain *x* is input into the feature extractor *F* to produce a feature vector $f_{x}=F\left(s_{x}^{t},a_{x}^{t}\right)$. *F* is trained to capture the structural similarities or the shared features between E and L domains by minimizing the distance between two features $f_{E}$ and $f_{L}$. Therefore, the loss function of *F* is defined as:

(4) $$\begin{array}{cc} L_{F}\left(F,G\right) & =E\left[∥F\left(s_{E}^{t},a_{E}^{t}\right)-F\left(s_{L}^{t},a_{L}^{t}\right)∥\right] \end{array}$$

(5) $$\begin{array}{cc} \; & =E\left[∥F\left(s_{E}^{t},a_{E}^{t}\right)-F\left(s_{L}^{t},G\left(F\left(s_{E}^{t},a_{E}^{t}\right)\right)\right)∥\right] \end{array}$$

### 4.2. Discriminator Network D and Generator Network G

The discriminator *D* is designed to distinguish between expert feature vector $f_{E}$ and learner feature vector $f_{L}$. Specifically, *D* receives a feature vector $f_{x}$ and outputs a probability $P(x=E|f_{x})$ to classify whether $f_{x}$ is from E or L. Meanwhile, the generator *G* aims to generate an action $a_{L}^{t}$ so that $f_{L}=F\left(s_{L}^{t},a_{L}^{t}\right)$ looks as similar as possible to $f_{E}$. In the proposed DAIL-GAN model, we apply adversarial loss [21] for both networks:   

(6) $$\begin{array}{cc} L_{GAN}\left(G,D\right) & =E\left[\log D(F\left(s_{E}^{t},a_{E}^{t}\right))\right]+E\left[\log\left(1D\left(F\left(s_{L}^{t},a_{L}^{t}\right)\right)\right)\right] \end{array}$$

(7) $$\begin{array}{cc} \; & =E\left[\log D(F\left(s_{E}^{t},a_{E}^{t}\right))\right]+E\left[\log\left(1D\left(F\left(s_{L}^{t},G\left(F\left(s_{E}^{t},a_{E}^{t}\right)\right)\right)\right)\right)\right] \end{array}$$

The optimal policy is achieved using a RL-based policy gradient, which relies on reward signal $r=-\log D(F\left(s_{E}^{t},a_{E}^{t}\right))$ provided by the learned discriminator.

### 4.3. Full Objective

During the learning phase, we aim to learn domain-shared features between E and L domains. Thus, the feature extractor *F* and the generator *G* are optimized to minimize the feature extractor loss $L_{F}$. At the same time, given a feature vector $f_{x}$ of domain *x*, we want to judge whether $f_{x}$ is from E or L by minimizing the domain classification loss $L_{GAN}$. This encourages domain-specific features to be captured by *F*. Overall, our full objective function is:

(8) $$\begin{array}{cc} \; & \underset{F,G}{max}\underset{D}{min}L\left(F,G,D\right) \end{array}$$

(9) $$\begin{array}{cc} \; & \;\mathrm{subject}\;\mathrm{to}\;\;L\left(F,G,D\right)=L_{GAN}\left(G,D\right)-\lambda L_{F} \end{array}$$

We wish to find a saddle point, where:

(10) $$\begin{array}{cc} \left(\hat{F},\hat{G}\right) & =\underset{F,G}{argmax}L(F,G,\hat{D}) \end{array}$$

(11) $$\begin{array}{cc} \hat{D} & =\underset{D}{argmin}L(\hat{F},\hat{G},D) \end{array}$$

At the saddle point, the $\hat{D}$ minimizes the domain classification loss. The feature extractor $\hat{F}$ and the generator $\hat{G}$ minimize the distance between both domains (i.e., the features are shared between domains), while maximizing the domain classification loss (i.e., the features are specific to each domain). The parameter λ controls the trade-off between domain-shared features and domain-specific features should be learned by *F*.

The algorithm of the proposed model is outlined in Algorithm 1.
**Algorithm 1:** DAIL-GAN1:**Input**2:    $D_{E}$  A set of expert demonstrations3:Randomly initialize feature extractor network *F*, generator *G* and discriminator *D*4:**for** i = 0, 1, 2, … **do**5:    Sample an expert demonstration $\tau_{E}^{i}∼D_{E}$6:    Update the parameters of feature extractor network *F* with the gradient
$E\left[\nabla_{F}log\left(D\left(f_{E}\right)\right)\right]+E\left[\nabla_{F}log\left(1-D\left(f_{L}\right)\right)\right]-\lambda E\left[\nabla_{F}∥f_{E}-f_{L}∥\right]$7:    Update the discriminator parameters with the gradient
$$E\left[\nabla_{D}log\left(D\left(f_{E}\right)\right)\right]+E\left[\nabla_{D}log\left(1-D\left(f_{L}\right)\right)\right]$$8:    Update policy $\pi_{L}$ with the reward signal $r=-logD(f_{E})$9:**end for**10:**Output**11:    $\pi_{L}$  Learned policy for learner domain

## 5. Performance Evaluation

In this section, the performance of the proposed DAIL-GAN model is evaluated by comparing with various baseline models on a number of tasks ranging from low to complex high-dimensional. The details of the experimental settings and evaluation results are presented in the following subsections.

### 5.1. Experimental Settings

#### 5.1.1. Environments

**Figure 3:**
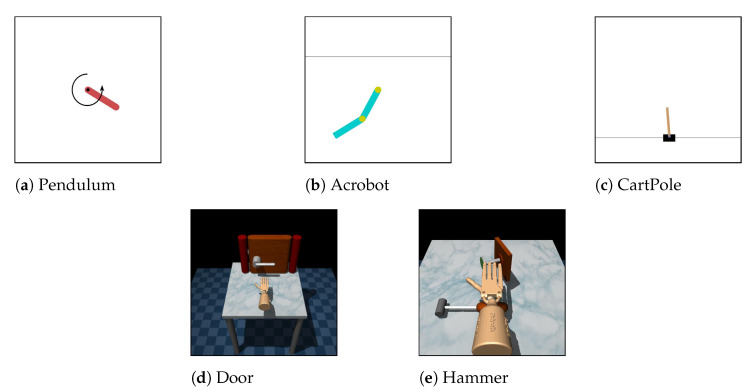
Visual rendering of five simulated environments used in our experiment.

In this experiment, five simulated environments were considered: Pendulum [37], Acrobot [37,38,39], CartPole [37,40], Door [41], and Hammer [41]. The detailed descriptions and visualizations of these environment are shown in Table 1 and Figure 3, respectively. From such environments, five domain adaptive tasks were decided, each of which included two different environments—an expert domain and a learner domain. These tasks can be divided into two categories as follows:

- Low-dimensional tasks:
  – Pendulum–Acrobot: Expert domain is Pendulum and learner domain is Acrobot.
  – Pendulum–CartPole: Expert domain is Pendulum and learner domain is CartPole.
  – Acrobot–CartPole: Expert domain is Acrobot and learner domain is CartPole.
  To provide expert demonstrations, for each task, the Trust Region Policy Optimization method [42] is first trained on the expert domain using the shaped reward signal. Then, 20 expert demonstrations are collected by executing the learned policies in the expert domain simulator. Each demonstration includes a sequence of state–action pairs. It should be noted that we only collect successful demonstrations where the learned policies can accomplish the task. The impacts of demonstrations on the performance of the proposed model will be analyzed in our future work.
- High-dimensional tasks:
  – Door–Door: The expert and learner domains have different friction parameters. The friction parameter in expert domain is [1,1,1], while, in the learner domains, it is [4.0,4.0,4.0].
  – Hammer–Hammer: The expert and learner domains have different mass of the hammer. The mass of the hammer in expert domain is 0.253442, while, in the learner domain, it is 1.0.
  We also use 20 expert demonstrations for each task. The demonstrations are collected from humans using the Mujoco HAPTIX system [43] and publicly available [41].

#### 5.1.2. Baselines

The performance of the proposed DAIL-GAN model was evaluated in comparison with the following baseline methods:

- Trust Region Policy Optimization (TRPO) [42] is a Reinforcement learning-based model. The model was trained directly on the learner domain and had access to the shaped reward function. This baseline set an upper bound for the performance of domain adaptation algorithms.
- GAMA-PA [14]: The model introduced a two-step approach for domain adaptation in imitation learning. It first learns the state–action maps between expert and learner domains, and then utilizes it to learn an optimal policy. The model parameters are employed as reported in [14] in order to ensure a fair comparison.

#### 5.1.3. Network Structure and Hyperparameters

Deep feed-forward networks with two hidden layers are used for three *F*, *G*, *D* networks of the proposed model. The network hyperparameters are shown in Table 2. In this experiment, the learning rate was 0.0003. Adam was used as an optimizer.

### 5.2. Results

In this subsection, the evaluation results of the proposed DAIL-GAN model on low- and high-dimensional tasks are presented to highlight its superior capability in domain adaptive imitation learning.

#### 5.2.1. Low-Dimensional Tasks

**Figure 4:**
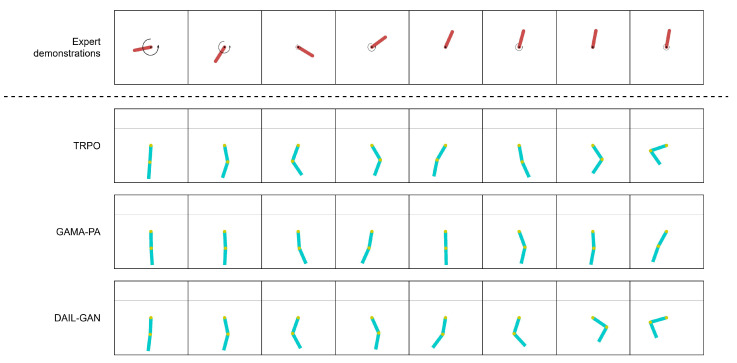
Pendulum–Acrobot.

**Figure 5:**
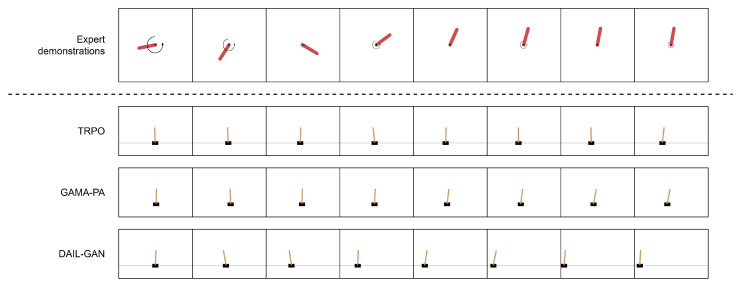
Pendulum–CartPole.

**Figure 6:**
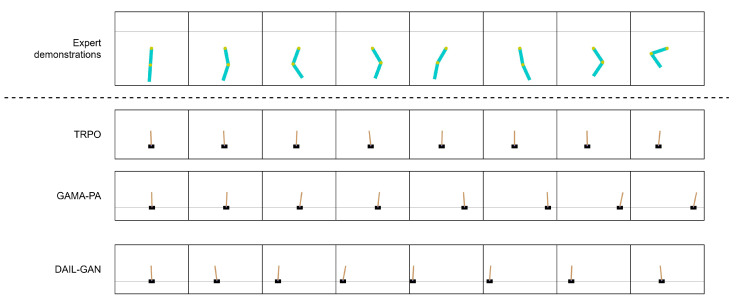
Acrobot–CartPole.

Table 3 reports the quantitative evaluations of the proposed DAIL-GAN model on low-dimensional tasks, in terms of average cumulative rewards. The numerical results clearly indicate that, for all evaluated tasks, TRPO [42] provided the best performance as its average cumulative rewards were at the highest. This was actually predictable because TRPO [42] had direct access to states and shaped rewards of the learner domain. On the other hand, inputs of GAMA-PA [14] and DAIL-GAN were limited to expert demonstrations only. As a result, their performances deteriorated compared to TRPO [42]. However, Table 3 also determines that the proposed DAIL-GAN outperformed GAMA-PA [14] across all three tasks. Additionally, for the Pendulum–Acrobot task, the proposed model almost achieved as high performance as TRPO [42]. In order to understand the observed results more deeply, Figure 4, Figure 5 and Figure 6 visualize the behaviors of learned policies provided by the evaluated models when performing the Pendulum–Acrobot, Pendulum–CartPole, and Acrobot–CartPole tasks, respectively.

In the expert demonstration of the Pendulum–Acrobot task in Figure 4 and the Pendulum-CartPole task in Figure 5, expert behaviors were to apply a strong force, expressed by a rotation velocity, at first to make the pendulum swing upright. After that, a few light forces were applied to maintain it vertically. Observing from Figure 4, the policies trained with GAMA-PA [14] failed to apply strong enough forces to swing the lower link as high as the proposed DAIL-GAN. In addition, Figure 5 expresses that the GAMA-PA [14] could not move the cart at an appropriate velocity to keep the pole vertical. We speculate that it was because the expert demonstration also did not show much movement after successfully swinging the pendulum upright as it only applied light forces. Meanwhile, the policies learned by our DAIL-GAN model could accomplish the task. Interestingly, we observed that the learned policies are able to produce behaviors that are relatively similar to the expert: the cart was first pushed to the left by a strong force; then, small forces are applied to prevent the pole from falling over.

For the Acrobot–CartPole task in Figure 6, the behaviors of the expert were that the link was swung back and forth to gain enough velocity to reach a higher height. Similarly, the GAMA-PA’s learned policy could move the cart faster compared to the Pendulum–CartPole task. However, it still failed to maintain appropriate velocity to keep the pole standing. On the contrary, our DAIL-GAN was able to remain the pole vertical. It is important to note that the learned policy of our model could move the cart in both directions, which is also similar to the expert behaviors.

The above observations show that the proposed DAIL-GAN model not only succeeded in imitating the expert behaviors but also adapted the learned policies well to a distinct learner domain. Meanwhile, although the GAMA-PA [14] could learn the state–action maps from expert to learner domain, its adaptation algorithm was inefficient to help it accomplish the tasks.

#### 5.2.2. High-Dimensional Tasks

**Figure 7:**
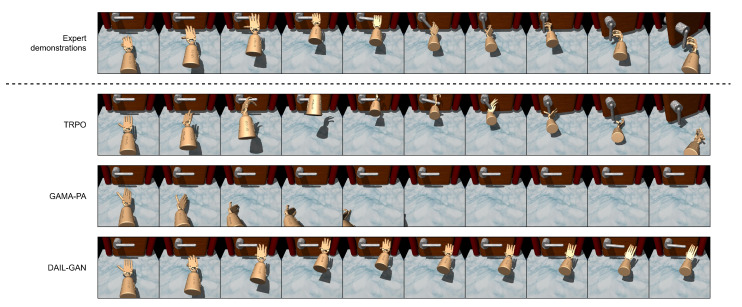
Door–Door.

**Figure 8:**
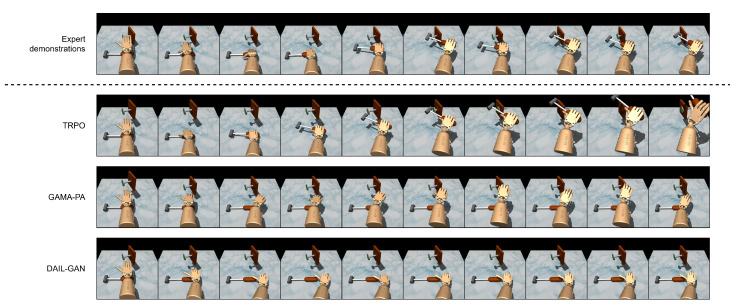
Hammer–Hammer.

In this subsection, the performance of the proposed DAIL-GAN versus the referenced models on the high-dimensional task is assessed. The average cumulative rewards of the evaluated models are shown in Table 4. As expected, the TRPO model achieved the highest average cumulative reward since it was trained directly on the learner domain. It is also revealed that DAIL-GAN outperformed GAMA-PA, although they were both unable to accomplish the Door–Door task. In addition, Figure 7 and Figure 8 depict the policies learned by TRPO [42], GAMA-PA [14], and our DAIL-GAN model, from which we observed some interesting behaviors.

As illustrated in Figure 7, the expert behaviors were understandable since their demonstrations were collected from humans: grab the handle, rotate it, then open the door. In Figure 8, the expert behaviors were to pick up and hammer multiple times in order to drive the nail into the board. While the policy trained with the TRPO could accomplish the task, it produced behaviors that were not human-like, i.e., unnatural use of the wrist to rotate the handle. The main reason behind these unnatural behaviors was that the TRPO depended on a careful reward shaping, and it was challenging to formalize human-like behaviors into a mathematical reward function. On the other hand, with the use of expert demonstrations, the GAMA-PA and the proposed DAIL-GAN were expected to generate human-like behaviors. However, the policy learned by GAMA-PA failed to control the hand properly, as shown in Figure 7 and Figure 8, due to the failure of the adaptation step in a high-dimensional task. Meanwhile, it can be observed from Figure 7 and Figure 8 that the policy trained with DAIL-GAN could produce more natural and human-like behaviors to move the robot hand closer to the door handle or the hammer. Unfortunately, our DAIL-GAN model could not rotate the handle or pick up the hammer in order to accomplish the task. Nevertheless, the human-like behaviors of the trained policies proved that our model could effectively extract and imitate expert behaviors from their demonstrations.

## 6. Discussion

This section discusses the overall performance of the proposed DAIL-GAN model, followed by the importance of the feature extractor.

The quantitative and qualitative results assessed from the previous section have shown the potential of the proposed DAIL-GAN model in tackling the domain adaptation problem in imitation learning. On both low- and high-dimensional tasks, DAIL-GAN could imitate expert behaviors from their demonstrations. In particular, the policies acquired by DAIL-GAN could even generate natural and human-like behaviors despite the high complexity of the Door–Door and Hammer–Hammer tasks. This indicates that the proposed DAIL-GAN could scale up to a complex manipulation task with a high-dimensional state and action space. Furthermore, the proposed model could adapt the learned policies to a distinct learner domain and accomplish low-dimensional tasks without being affected by the presence of domain shift between expert and learner domains. Although the success rate remained limited and depended on the complexity of the tasks, the proposed model can be improved to provide a better performance toward practical real-world imitation learning tasks.

The promising performance of the proposed DAIL-GAN also praises the effectiveness of the proposed feature extractor *F*. The feature extractor aims to learn both domain-shared and domain-specific features between expert and learner domains. In Figure 5, the learned policy tended to move the cart to the left by a strong force initially, then followed by small forces; this behavior was similar to that of the expert demonstration. Such a similarity indicated that the feature extractor could extract the structural similarities or domain-shared features between expert and learner domain, resulting in comparable behaviors between them. Furthermore, it can also be observed in Figure 5 that, although strong forces were applied, the learned policies still managed to keep the pole stay upright. This showed that the feature extractor was able to learn the differences between the expert and learner domains so that it could adapt the learned policies to the learner domain and accomplish the task. In summary, the feature extractor has proven its important role in our model. It could acquire shareable behaviors in both domains by learning the domain-shared features and adapting those behaviors to the learner domain regardless of the domain shift by learning the domain-specific features.

## 7. Conclusions

In this paper, we proposed a novel model for domain adaptive imitation learning, in which a feature extractor was introduced to learn the domain-shared and domain-specific features. The comprehensive evaluation on both low and high-dimensional tasks demonstrates that the policies learned by the proposed model can imitate expert behaviors and adapt them to a distinct learner domain. Thus, the potential of our proposed model and the effectiveness of the feature extractor were verified. In future work, we intend to extend the proposed model to improve its performance on more complex real-world imitation tasks.

## Figures and Tables

**Table 1:** Description of five simulated environments used in our experiment.

Task	State Space	Action Space	Description
Pendulum [37]	3 (continuous)	1 (continuous)	Swinging up a pendulum.
Acrobot [37,38,39]	6 (continuous)	3 (discrete)	Swinging the end of the lower link up to a given height
CartPole [37,40]	4 (continuous)	2 (discrete)	Preventing the pendulum from falling over by applying a force to the cart.
Door [41]	39 (continuous)	28 (continuous)	A 24-DoF hand attempts to undo the latch and swing the door open.
Hammer [41]	46 (continuous)	26 (continuous)	A 24-DoF hand attempts to use a hammer to drive the nail into the board.

**Table 2:** DAIL-GAN hyperparameters used in the experiment. Each number corresponds to the number of nodes in a network layer.

	Feature Extractor *F*	Generator *G*	Discriminator *D*
Low-dimensional Tasks	$\left(s_{x}^{t},a_{x}^{t}\right)$ - 32 - 32 - 16	$\left(f_{x}\right)$ - 32 - 32 - $\left(a_{L}^{t}\right)$	$\left(f_{x}\right)$ - 32 - 32 - 1
High-dimensional Tasks	$\left(s_{x}^{t},a_{x}^{t}\right)$ - 128 - 128 - 64	$\left(f_{x}\right)$ - 128 - 64 - $\left(a_{L}^{t}\right)$	$\left(f_{x}\right)$ - 128 - 64 - 1

**Table 3:** The performance of the proposed models on low-dimensional tasks. These scores represent the cumulative rewards obtained from executing a learned policy in the simulator, averaged over 100 episodes.

Task	TRPO [42]	GAMA-PA [14]	DAIL-GAN
Pendulum–Acrobot	−63.18 ± 7.05	−386.31 ± 49.20	−83.31 ± 32.61
Pendulum–CartPole	497.13 ± 28.56	144.03 ± 89.09	289.74 ± 171.21
Acrobot–CartPole	497.13 ± 28.56	93.05 ± 88.97	153.86 ± 81.79

**Table 4:** The performance of the proposed models on high-dimensional tasks. These scores represent the cumulative rewards obtained from executing a learned policy in the simulator, averaged over 100 episodes.

Task	TRPO [42]	GAMA-PA [14]	DAIL-GAN
Door–Door	2449.06 ± 1175.25	−65.19 ± 0.77	−33.51 ± 8.87
Hammer–Hammer	17,030.25 ± 4357.23	−252.52 ± 4.91	−78.84 ± 19.28

## Data Availability

Publicly available datasets were analyzed in this study. This data can be found here: https://sites.google.com/view/d4rl/home (accessed on 1 July 2021).

## Abbreviations

The following abbreviations are used in this manuscript:

RL	Reinforcement Learning
IRL	Inverse Reinforcement Learning
GAN	Generative Adversarial Network
BC	Behavior Cloning
MDP	Markov Decision Process
